# The continuing formation of relational caring professionals

**DOI:** 10.1007/s11019-022-10104-0

**Published:** 2022-08-27

**Authors:** Guus Timmerman, Andries Baart

**Affiliations:** 1 Presence Foundation, Grebbeberglaan 15, 3527 VX Utrecht, The Netherlands; 2 University Medical Center Utrecht, PO Box 85500, 3508 GA Utrecht, The Netherlands; 3 Optentia Research Focus Area, North-West University, Vanderbijlpark, South Africa

**Keywords:** Formation, Relational caring, Care ethics, Exposure, Continuing education, Case-based learning, Practical wisdom

## Abstract

Learning to work as a relational caring professional in healthcare and social welfare, is foremost a process of transformative learning, of Building, of professional subjectification. In this article we contribute to the design of such a process of formation by presenting a structured map of five domains of formational goals. It is mainly informed by many years of care-ethical research and training of professionals in healthcare and social work. The five formational domains are: Relational Caring Approach, Perception, Knowledge, Interpretation, and Practical Wisdom. The formation process, described as the recurring detour of a continuing practice, requires ‘exposure’, in-depth learning and learning communities. Relational caring—care consequently resulting from and structured by relational thinking, exploring, and steering—requires ‘inquiry’ as a continuous learning process in practice. The process is ultimately aimed at fostering mature, competent, and practically wise professional caregivers who are able to relationally connect with and attune to care receivers, and adequately navigate existential, moral, and political-institutional tensions in relational caring in complex organizations in Late-Modern society.

Although it is difficult to imagine care that is non-relational, relational caring—i.e. care consistently resulting from and structured by relational thinking, inquiring, and steering—is something all together different. It is one thing to confess that care is relational, it is something different to practise it, think about it, steer it in a relational way. Relational caring, as we understand it, is the core or the heart of all good professional care, help and support, from the everyday face-to-face care provided by nurses or the social work in neighbourhoods and playing grounds to the highly specialised care provided by a brain surgeon or by youth workers in closed facilities for special youth rehabilitation treatment. It can indeed be found in care practices in healthcare and social welfare and there are care professionals who do indeed practise presence and relational caring (Baart 2001, 2018b; Baart and Vosman 2011, Timmerman and Baart 2016b).^1^ However there are also many professionals who find it too difficult, who think it will take more time than they have available, who are unable to see how to incorporate it into what they consider to be their profession, or who consider it to be something just for virtuosos. This article deals with the question of how it can be learned. And what is needed to design and organize educational curricula to foster relational caring professionals (Baart 2014). And what is needed for the continuing education of relational caring professionals. It is an empirically informed attempt to zoom out from (educational processes aimed at) the different professional practices and professions in healthcare and social work, while mediating between (empirically grounded) theory and practice (in care practices and education), between care (in healthcare and social work) and (strong) relationality, and between (a political take on) care ethics and (critical-reflexive) *Bildung*.

Our proposal is based on the learning and transition processes we have organised, studied, initiated, implemented, and evaluated over the past 20 years (Baart 2001, pp. 209–282; Baart et al. 2011; Baart et al. 2015; Leest et al. 2018; Beurskens et al. 2019; Bontemps-Hommen et al. 2020). Learning to practise presence and relational caring requires learning processes that differ from those required for learning to apply theories and follow methods. Engaging in ‘exposure’, being trained in open and attentive perception, learning from exemplary practitioners, changing perspective, practising case-based learning, watching movies together and participating in learning communities have an effect primarily on attitude, understanding, inquisitiveness and other qualities of the person, and only secondarily on knowledge and skills. There is an existential aspect to relational caring and, therefore, to the education of relational caring professionals: one must understand something about life and the fragility of human existence and be aware of what it is like to be ill, lonely, poor, abused, physically impaired, dependent, silenced, a person with dementia, made redundant. Usually, there are many reflexes, habits and routines which need to be un-learned: labelling, knowing what’s the case in advance, jumping to a solution, shying away from what is unknown or too complex. And, interestingly, the persistent tendency to add rather than subtract components in attempts to solve a problem (Adams et al. 2021). What is always fostered in our learning processes is solicitude, a genuine commitment to people in need of care and support. Our moral conviction is that the other person (the client or patient) must be recognised and acknowledged as a member of the one human family and, as a consequence, deserves relational and social inclusion. Many of the other normative claims we make—concerning perceiving, connecting with and attuning to the other, different sources of knowledge, et cetera—are a non-foundational consequence of this fundamental moral conviction.

Our insights have been developed through many years of training and supervising, and in an ‘oscillating’ movement between empirical research, theory development and ethical reflection (Baart 2020). We position ourselves in the literature about the developing discipline of care ethics as an empirical endeavour (Klaver et al. 2014; Leget et al. 2017; Vosman et al. 2018), within the framework of a political take on care ethics (Vosman 2020), viewing care as a moral practice, in particular within the framework of presence theory (Baart 2001). We draw heavily on discussions about the reflective practitioner (Argyris and Schön 1974; Schön 1983, 1987), normative professionalization (Jacobs et al. 2008; van Ewijk and Kunneman 2013), *Bildung* (Klafki 2007) and practical wisdom (Bontemps-Hommen 2020; Kinsella and Pitman 2012; Schwartz and Sharpe 2010). Our proposal should be situated in the context of comprehensive transitions in care and care organizations (Loorbach and Rotmans 2010) in Late Modernity, in the domains of both healthcare and social work. Late modern circumstances—especially complexity and the need of complexity reduction, and precariousness, taken as the installment of uncertainty—require a rethinking of the predicament of care and care organizations (Vosman and Niemeijer 2017). As a contribution to these transitions, the education of relational caring professionals needs to involve in-depth learning, and professionalization as a ‘counter programme’, i.e. an alternative professional way of giving care (Daaleman et al. 2011). Our proposal is ultimately aimed at fostering mature, competent, reflexive and practically wise professional carers who can relationally connect with and attune to (groups of) care receivers, and who can adequately navigate existential, moral, and political-institutional tensions in relational care within complex organizations in Late-Modern societies.

## Formation process

Learning to work as a relational caring professional is not only a process of vocational qualification—acquiring technical, communicative, and attitudinal competences—or professional socialization—becoming a valued member of the community of professionals. It is also and foremost a ‘formation process’: a process of transformative learning (Laros et al. 2017), of *Bildung*, and especially critical-reflexive *Bildung*, aiming at a ‘values-driven transformation of both individual learners and society’ (Sjöström et al. 2017), of professional subjectification (Biesta 2020). Whereas qualification is about acquiring the knowledge, skills, dispositions and competences of the profession, formation is also about ‘forming’—i.e. the personal development of—the professional to become a mature, sensitive, inquisitive, responsible person, familiar with and able to understand and navigate existential, moral, and political issues, in a professional position in society. Because giving and receiving care are political activities, constitutive of the *polis*, such a position is also a political position (Vosman 2020). Ultimately, the formation of the relational caring professional is not only about the sustainable development and (self-)transformation of the professional, but also of professional practice, care organizations and society as a whole.

Every professional needs to master the necessary knowledge, skills, dispositions, and competences of their profession. However, a process of formation is needed to prevent the student from developing an instrumentalist attitude to these resources (Reichenbach 2014). Because in relational caring the professional’s self is their most important ‘instrument’, practising being *a* self—i.e. the subject of one’s own life and one’s own acting, open to the appeal that is being made, and free to respond (or refrain from responding) to that appeal—is the core of their formation. This core is what Gert Biesta calls ‘subjectification’. It is about encountering one’s freedom when engaging a ‘reality check’ or encountering responsibility (Biesta 2020). Moreover, relational caring is not just a method, a set of methods or a system of methods, but an approach—i.e. a comprehensive way of perceiving, reflecting, evaluating, being courageous, doing and refraining, using artefacts, and knowing—within the framework of a substantial idea of good care. Whereas a method is an effective procedure in which a particular, pre-given goal can be achieved with a degree of certainty by undertaking certain steps in a specific order, an approach is conjectural; the practitioner must do everything possible to achieve the emerging goal but can never be sure in advance that he or she will succeed (Baart 2001; 2018b). Therefore, the formation of the relational caring professional is not only about learning methods but also about becoming familiar with, appropriating and becoming able to criticize from within the underlying epistemology, ontology, axiology, and praxeology of relational caring. Because of the nature of such an approach learning to practise relational caring requires a formation process, and without continuing such a process the professional will likely cease to practise relational caring. Because relational caring is an approach and the person of the professional is essentially involved, the qualities the formation process is aiming at are qualities of the person and not simply ‘competences’, to be acquired by the person.

There are a lot of criteria for the success of such a formation process, but they have to be relationally assessed. In our own training and formation processes, most of these criteria are being assessed in terms of more or less: understanding, ability to perceive openly and inquisitively, reflection, clarity about what one’s profession is about, awareness of the scope for action, management of relationships, actually trying out new behaviour, et cetera. There is a small, but essential cluster of criteria that is assessed dichotomous. Change of perspective, determining in the relationship which good should be pursued, letting the other emerge, et cetera, are not a matter of more-or-less but of yes-or-no. This cluster is about putting relationships at the centre for the good of the client or patient and is essential to relational caring. In addition, when a whole team is involved in a formation process, the room for joint reflection and the experienced benefit to clients or patients and their relatives in the care of this team are also assessed.

## A map of core formational domains

**Fig. 1:**
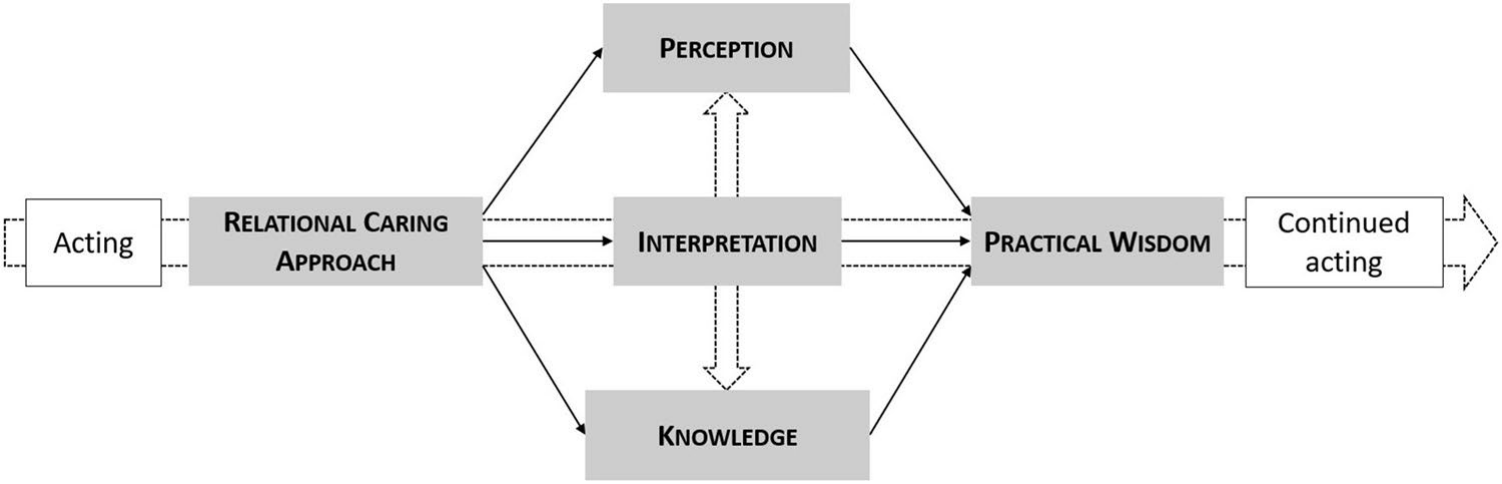
The five core domains of the formation of relational caring professionals

Rather than proposing an elaborate model of professional formation, this article presents a map of five domains of formation, including related theoretical stances and preferences, together with formational goals for each of these domains: Relational Caring Approach, Perception, Knowledge, Interpretation, and Practical Wisdom (see Fig. 1). It is essential to understand that this map is not a representation of the acting of the professional or the practising of relational caring, nor is it a model of how to organize the formation process. It is not a trajectory of formational goals, nor a unidirectional process of causes and consequences. It is a map of the domains of the main formational goals that need to be included in the process of forming a relational caring, presence practising professional. And the map is not simply a list; the domains are arranged in a deliberate way, with a direction from left to right and a vertical interplay. Both the domains as their arrangement are special and unusual.

Central in the map is a horizontal arrow from left to right, expressing the empirical truth that any development process is embedded in an ongoing practice. There is always a way of doing and learning before and after the formation process, which is institutionalized in protocols, methods, and artefacts as well as in taken for granted habits of caring, reflecting and collaborating. This embedment of the formation process ensures the continuity of care but at the same time is also a source of resisting forces, repelling attempts to change it. Being the recurring detour of a continuing practice, the formation process therefore encompasses not only learning but also un-learning and re-learning. At the left of this arrow is Relational Caring Approach, the all-encompassing philosophy of relational caring which imbues all the other domains. By calling it an approach, we distance it from the (false) idea that care is a method, that following specific steps in a specific order will necessarily result in specific outcomes. At the right of the arrow is Practical wisdom, needed to act in situations of uncertain outcomes, conflicting guidelines, and contradictory goals.

Also central to the arrangement of the map is a vertical interplay between perception and knowledge. Perception is about how the professional perceives the situation, the relationships in the situation, the patient or client (with their vulnerabilities, needs and concerns), their own position (including power relations), the care organization and society. Knowledge in this vertical interplay is about the knowledge and know-how the professional has access to and is supposed to use, including patient and family knowledge. It is an interplay because all perception is theory laden and what is perceived determines what knowledge is activated. At the intersection of these two movements is Interpretation, the determination of the meaning, relevance, and significance of what is at stake and of what can and cannot be done by the professional.

### Relational caring approach

**Fig. 2:**
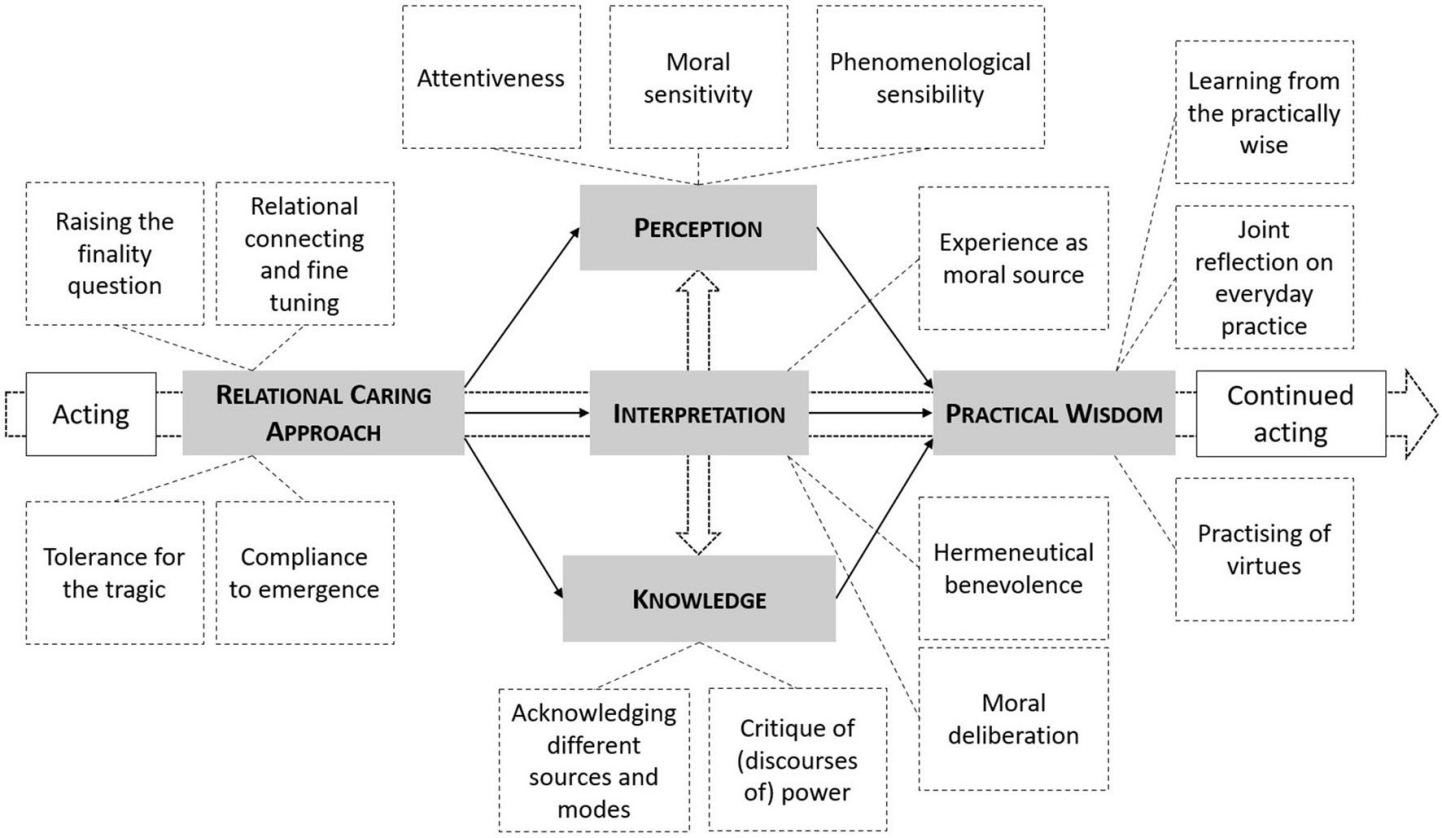
Detailing the goals of the formation of relational caring professionals

The formation of relational caring professionals involves a familiarization with the way in which care ethics and presence theory conceptualize and theorize healthcare and social work, namely as a practice of relational caring. Healthcare and social work are practices, each consisting of a highly complex interplay of practitioners and other participants in the practice, doings and sayings, refrainings and undergoings, artefacts, bodies, habits, protocols, physical environments, machines, spaces et cetera. The individual professional is decentred: without nullifying their agency, the doings of a practice ‘befall’ the participants in the practice (Nicolini 2012; Schatzki 1996). Next, healthcare and social work practices are about caring, not about solving problems or implementing interventions as such, nor about producing commodities or delivering services. Lastly, healthcare and social work are about caring for patients and clients who are suffering, undergoing both their predicament and the care practice (Vosman et al. 2016). The formational goals in this domain contribute to understanding the importance of, the being able to and having the willingness to: (a) raise the finality question, (b) relationally connect with and attune to the care receiver, (c) tolerate the tragic aspects of life and (d) comply with emergence (see Fig. 2).

The questions ‘What is care (here: covering healthcare and social welfare)? What is care about? What is good care?’ are concerned with the *telos*, the end, the purpose, the ultimate goals, the aim, the tenor, the finality of care as a practice (Nicolini and Monteiro 2017). Knowing what the purpose, the telos, or the finality of care is, is helpful in finding out what to do or to refrain from doing in a particular situation with a particular patient or client and their relatives. And to handle the valid rules in situ. Raising the ‘finality question’—What is this really about? What is it that really motivates us the most in this work? What is the contribution of this to society? What exactly is the ultimate purpose of this?—helps to contribute to good care, to make scope for practical wisdom, and to install a basic frame of reference for perceiving, interpreting, acting, refraining, and evaluating within a particular practice, type of work, organization, or institution. Answering the finality question is a joint effort to situationally articulate an answer for the time being. Its outcome is a discursive performance, open to future revision. And it requires formation.

Good care requires a sustained effort to radically connect with and attune to the care receiver in language, capacity and knowledge, but also in movement, tempo and rhythm (Baart 2001). Relational caring professionals need to be attentive to, and able to understand the meaning and significance of, the way the care receiver moves, physically, emotionally, and socially, what they find attractive or repulsive, what they move towards and what they move away from. Phenomenologically speaking, the professional falls into sync with the other in his or her life (Waldenfels 2004), and tries to figure out what is at stake: in the centre, in the background and underneath the surface. What is at stake is not necessarily what is problematic. From moving to being moved is the essential step towards relational caring by the professional (Baart 2011). The good to be pursued is sought and determined from the perspective of relationships and is not selected or prescribed by the discipline, the applicable policy, the institution, et cetera, whether or not neatly dressed up in a relational manner. The professional, however, is not necessarily moved by everything the other deems important. There is always a need for the professional’s own decision about what to attune to, attuning also to their own abilities and limitations, their colleagues and the institutional contexts. Conflicts do not have to be avoided, as long as they are navigated in a relational way. Taking care of existing relationships and finding out in the relationship what is at stake for the other are part of it, the ability to terminate a relationship without abandoning the other also. This is where finality and practical wisdom come in.

Care does not always solve problems, cure patients, help clients, improve someone’s lot. Life is frail and complex and, although it can be manipulated, a desirable outcome of an intervention cannot be ensured. Professionals must have the ability to perceive, understand, endure, and relate to (and not flee from) hopelessness and the tragic failure of care. They know and have integrated within themselves the notion that, when nothing further can be done, one can still stay and be with the other (van Heijst 2011). Relational caring is more like charitably and sensibly ‘muddling through’—meandering, continuously learning and adjusting, always with the *telos* of care in mind—than like straightforwardly fixing problems according to a predetermined plan (Baart 2013; Lindblom 1959, 1979). In the context of ‘broken goods’, i.e. results that are not in all aspects ‘good’ although one is successful (Baart 2004), relational caring needs to be carried out with dedication. Tolerance of the tragic is an indispensable goal in the formation of relational caring professionals.

Care is the intersection of multiple practices and as such highly complex, unstable, and emergent (Vosman and Niemeijer 2017). Relevant variables may be unpredictable, non-reducible and untraceable (Greve and Schnabel, 2011; Morin 2008). Professionals must be trained to be open to and receptive of this complex, dynamic, multivocal, unstable and emergent character of care, instead of becoming social-technical operating functionaries with a communicative topping.

The formation process aims at acquiring and developing a basic attitude of thinking about healthcare and social welfare from a relational caring perspective, the ability to recurrently and jointly raise and answer the question of what the practice is about, and an awareness of and ability to navigate complexity, dynamics, and emergence. Their tolerance for the tragic will have been fostered.

### Perception

According to political theorist and care ethicist Joan Tronto, the first phase of care is ‘noting the existence of a need and making an assessment that this need should be met’ (Tronto 1993, p. 106). This need has first of all to be perceived, and this requires an open, receptive, and inquisitive way of perceiving. This perception of need is identified in interaction with the professional’s knowledge and leads to an interpretation that is meaningful and sensible (Sayer 2011). The formational goals in this domain are related to the development of competent, appealable (a) attentiveness, (b) moral sensitivity and (c) phenomenological sensibility (see Fig. 2).

Perception of someone’s neediness requires attentiveness, which is both instrumental and in itself beneficial. The other person is taken seriously (Arvidson 2006; Klaver and Baart 2011; Klaver 2016). Relational caring professionals are interested in what is at stake in the life of the care receiver, in their ‘concerns’ at that moment (Olthuis et al. 2014). The focus, scope and wider context of the caregiver’s attentiveness determines the answer to the question: to whom and what is relational caring aimed at, what do caregivers consider and what do they not consider their task and responsibility? This involves a process of inclusion and exclusion that is continuously adjusted, narrowed down, and expanded during the caring process. This attentiveness implies more than being concentrated and alert, it mainly has to do with being interested in the care receiver, their perspective and what is at stake for them. Watching TV documentaries can be particularly revealing and also painful in this respect. In that interested attentiveness, relevant themes present themselves. This interplay of foreground and background, centre and periphery, focus and margin is what determines the caregiver’s agenda and playing field. It is not a stable whole because shifts are constantly taking place between the centre and the periphery, themes are developing and gaining or losing importance (Timmerman and Baart 2016a). Being attentive is an act of giving, it constitutes a relationship, and it is the beginning of social inclusion and recognition. And it must be learned and constantly refined.

Making an assessment that the perceived need should be met is a moral activity that requires sensitivity to the appeal of the other. This sensitivity prevents the professional from just turning away from the other in need. It requires developing the willingness and capability to (i) marvel at and be open to unexpected meanings (Arvidson 2006; Senge et al. 2005; Verhoeven 1972); (ii) be receptive to appeals and to change between proximal and distal consciousness, between focal and subsidiary attentiveness (Baart 2008; Polanyi 1966); (iii) take the ‘perspective from within’ on indigence (Housset 2003).

Care is about concrete needs, experienced from the first person’s point of view. Therefore, perception requires a phenomenological perspective, aimed at finding out how things manifest themselves to the care receiver; how they present themselves in experience and thus come to have meaning. Methodologically, this means learning to (i) practise bracketing, bridling, or managing one’s own preconceived meanings, expectations, et cetera (LeVasseur 2003); (ii) become aware and critical about modern ‘social imaginaries’ (Taylor 2007), and finally (iii) be able to reconstruct the quintessence of the phenomenon in the receptivity of the care receiver (Benner 1994; Vosman 2018). Regularly engaging in exposures is very helpful in this respect.

The formation process aims at acquiring and developing the ability to perceive the care receiver and oneself as freely, openly, and carefully as one can, and attentiveness, moral sensitivity, and phenomenological sensibility.

### Knowledge

Professionals lean heavily upon all kinds of professional, discipline-specific knowledge. In the complex reality of relational caring in healthcare and social work, there are different sources and modes of knowledge, and a power struggle about which and whose knowledge should have the say regarding what should or should not be done. The formational goals in this domain contribute to being able to: (a) acknowledge different sources and modes of knowledge and (b) criticize (discourses of) power (see Fig. 2).

Usually, the professional knowledge of the professional has priority over the knowledge of other parties, especially that of the care receiver and their relatives. The formation of the relational caring professional should aim at making room for (i) the informal insights of the care receiver and their relatives, for example about what will work with the care receiver; (ii) their semiotic interpretations based on familiarity, for example their understanding of the meaning of the care receiver’s behaviour, posture and facial expression against the background of their shared life and history, and (iii) their moral preferences and boundaries, for example about what they regard as morally appropriate concerning the continuation of the treatment. Making room for these three types of knowledge curbs the ‘epistemic paternalism’ of the professional, diminishes ‘epistemic injustice’ and promotes a more just distribution of ‘epistemic authority’ (Brister 2012; Fricker 2007). Being aware of cultural differences is conducive to promoting ‘cognitive justice’ (de Sousa Santos 2018).

A related issue is the differing status of the various professionals and their professional knowledge. For example in a hospital there can be conflicts between the epistemic perspectives of nurses, attending physicians, medical specialists and directors on the treatment of a particular patient. This hierarchy of knowledge sources and modes must be navigated situationally, in a joint deliberation. In addition to discretionary space for professionals, there must be ‘systemic space’ (Vosman 2008): space within the system in which various practitioners within the organisation can jointly consider the finality of their crossing practices and find out what that means for what they have to do in the case of this particular patient.

Our political take on care ethics makes us aware of the power of knowledge, and of the institutions and societal structures in which people are being cared for (Barnes 2012). We distinguish between the local, first-level discourse of power, situated in concrete care practices, and the more general, second-level discourse of power, situated in politics, which governs for instance the (taken for granted) distribution of care and caring responsibilities. The second discourse ‘encompasses’ the first and is influential as the embedding politics of caring. In concrete care practices, there are already deeply granted powers in effect, disciplining the acting of the individual participants. These powers take the shape of compulsory procedures, rules, and routines, but also of people in certain positions with whom the professional must interact. In the distribution of care and caring responsibilities, a differentiated society is reproduced in which certain categories of people deserve care and others do not. And in which certain categories of people are predominantly caring while others claim to be safeguarded against the responsibilities of caring (cf. ‘privileged irresponsibility’, Tronto 1993). This distribution of care and caring responsibilities is internally linked to the distribution of power, precarity and recognition (Baart 2021).

The formation process in this domain aims at acquiring and developing the ability to access and bring into play different sources and modes of knowledge, a critical awareness concerning the discourses of power on different levels, and a certain resilience (Benard 2004).

### Interpretation

At the intersection of perception and knowledge, interpretation looks for ‘understanding’ and for the expansion of the ‘horizon of understanding’ (Gadamer 1994). Here, again, it is important to understand that care is a moral practice; it is not a morally neutral phenomenon in need of ethical principles only when confronted with ‘ethical dilemmas’. The formational goals in this domain contribute to the development of the ability to: (a) practise hermeneutical benevolence, (b) recognize and work with emotions as moral intuitions, and ‘ethos’ as a source of ethics and (c) use moral deliberation (see Fig. 2).

Professionals need to be able to treat everyone impartially, also those who are different from the people with whom the professional is familiar. A certain degree of benevolence when interpreting the other is needed. Hermeneutical benevolence has at least three aspects. The first is an interested attentiveness to the other that does not expropriate the concrete specificity and uniqueness of the other by categorising and ‘othering’. The second is the assumption that in the other, their lives and what is at stake for them, there is an interesting, rich meaning that one is not able to immediately understand. And that there will always remain a meaning surplus in it, that still must be understood. Full understanding is impossible, caution and reticence are always necessary. The third aspect of hermeneutical benevolence is the idea that this particular meaning and significance for the other has moral relevance to the professional. It assigns a position to the professional: to be the other’s advocate, helper, friend, or guest. The role of the professional is ‘inscribable’, that is: the care receiver may decide ‘who’ the carer is to them, mostly in a metaphorical way (Baart 2001).

According to cognitive theories, emotions inform moral intuitions: they tell us what is abhorrent and wrong, which values are hurt, what is regrettable (Nussbaum 2001, 2004, 2013; Roeser 2011). They encourage us to say ‘no’, not to go any further, to stop. They tell us that something is wrong and unjust, who is (undeservingly) a victim, what should be done or refrained from. Emotions can be interpreted as moral intuitions without asserting that they are infallible (van Tongeren 1999). Of course, moral intuitions can be misguided, rooted in controversial sentiments and unjust evaluations (Baart 2013). But at the same time, they can also be a critical moral memory breaking through the bureaucratic regime of rules, procedures, and institutionalized cruelty and neglect. We do not know what they are beforehand and that is why they should be morally explored and criticized (Dancy 2017).

What is good is what empirically turns out to do good. Therefore, empirical analysis is indispensable in moral deliberation. Based on the distinction of three types of feelings—sentiments, emotions and affects—one can distinguish between three types of moral deliberation (Baart 2013). The first is the ideology critique of sentiments, society’s feelings about categories of people, be it migrants or babies, and ‘social imaginaries’ (Engster 2007; Sevenhuijsen 2004). The second is the critical hermeneutics of moral experiences (van Tongeren 1994, 1999), and the third is the critical investigation into defendable moral particularism (Dancy 2017).

The formation process in this domain aims at acquiring and developing a critical awareness of the inherently moral aspects of relational caring, hermeneutical benevolence, the ability to explore and manage emotions as criticisable moral intuitions, and ethos as a criticisable source of ethics, and the capability to use different types of moral deliberation.

### Practical wisdom

Sometimes, acting is preceded by making a judgement and taking a (series of) decision(s), in which perception, knowledge and interpretation come together and that leads to an intention to act in a specific way. More often, acting is done while perceiving, reflecting, consulting knowledge, considering, interpreting, assessing, weighing, acting, and evaluating. Especially when one must act in case of conflicting goals, contradictory rules and uncertain outcomes, practical wisdom is a more helpful concept than judgment or decision alone. This is even more the case in Late Modernity in which health and social care is given in a society and in organizations characterized by complexity, ambiguity, and systemic pressure (Vosman and Niemeijer 2017). The formational goals in the domain of practical wisdom are related to: (a) learning from practically wise persons when and how to act in a practically wise way, (b) engaging in joint reflection on everyday practice by practitioners focused on acquiring practical wisdom and (c) the training of (other) virtues like parrhesia, dedication and tenacity (see Fig. 2).

Professionals have all kinds of formal, explicit, and codified knowledge, among which knowledge of proven interventions learned from books, transferred in schools, or found in databases. Advanced professionals also have their experience, their embodied know-how (Sennett 2008), their tacit knowing (Polanyi 1958, 1966). Tacit knowing is the result of what one has gone through in life, what one has perceived of what experienced colleagues have shown, what one has learned from what went well and what did not. Using explicit knowledge is always embedded in tacit knowing. Acting however, requires practical wisdom: the ability to discern what might be morally good and to make morally sound decisions. Not exclusively based on explicit, intellectually motivated reasons but also based on one’s own mature, moral experiences and the wisdom to use those experiences in complex and non-standard situations (Aristotle 2013; Kinsella and Pitman 2012; Timmerman and Baart 2016a; Vosman and Baart 2008). Learning how to act in a practically wise manner can be achieved by studying the way experienced, advanced, practically wise colleagues act.

There is some discussion in the literature about whether practical wisdom can only implicitly and individually be learned by practising itself or also be acquired through intentional learning processes in groups (Kinsella and Pitman 2012). Recent research has shown that physicians in hospitals can acquire and develop practical wisdom in their practices through regular, learning-oriented case discussions (Bontemps-Hommen 2020; Bontemps-Hommen et al. 2020).

In difficult situations, where the temptation is to end the caring process notwithstanding the evident neediness of the care receiver, three specific virtues are required to support practical wisdom. The first virtue is the courage to speak the truth in the face of danger, to say what one has to say regardless of the consequences for oneself. It is called ‘parrhesia’ (Foucault 2001, 2011). The second virtue is ‘dedication’, a complete and wholehearted loyalty to take care of the other, whatever it requires (Grøthe et al. 2015). The third virtue that is needed, is ‘tenacity’, the strength not to give up, choose the easiest path, or select only the promising patients or clients, but to stay and persistently search for the good of the care receiver, however difficult, nasty, or unthankful they may be (Kuokkanen and Leino‐Kilpi 2001).

The formation process in this domain aims at acquiring and developing a critical awareness of the relevance for acting of virtues in general and practical wisdom in particular. Practical wisdom will have been fostered by learning from practically wise colleagues and engaging in joint reflection on everyday practice focused on practical wisdom. Virtues like parrhesia, dedication and tenacity will have been nurtured.

Returning to the issue of the professional position as a *political* position, we just mention which domains, in our view, certainly address it. The contribution to society of one’s profession and institution is considered in raising the finality question. The political ideology of the socially engineered society is kept at a distance by the tolerance for the tragic. The entanglement of caring and knowing with power in its different shapes is made aware and criticised in the Knowledge domain. Finally, because of its connection with the finality of one’s professional practice, the domain of Practical Wisdom is relevant to training professionals in navigating moral and political tensions.

## Formational resources

So much for the ‘what’ of learning to practise presence and relational caring, we now turn to the ‘how’, based on our experience with educating, training and supervising professionals and teams of professionals in healthcare and social welfare (see Table 1). Some of these formational resources are quite unusual. The formation process requires at the very least exposure, inquiry, deep learning and learning communities. It is embedded in ongoing practice. Case-based learning and cultivating quality awareness contribute to the formation process. Art may be helpful. Essential is that care organizations facilitate permanent learning by providing time, resources, skilled facilitators, and a culture in which professionals can freely show themselves and tell about their fears, doubts and mistakes.

**Table 1:** Substantiation, exemplification and justification from our own research and supervision processes: the most important sources

Sources → Domains ↓	Baart (2001)	Baart and Grypdonck (2008)	Baart et al. (2011)	Baart (2013)	Baart et al. (2015)	Timmerman and Baart (2016)	Leest et al. (2018)	Baart (2018)	Beurskens et al. (2019)	Bontemps-Hommen et al. (2020)	Du Plessis and Beurskens (2021)
Formation and learning			pp. 103–200		pp. 269–289		pp. 72–92		pp. 159–175		
RELATIONAL CARING APPROACH	Passim			Esp. pp. 9–16, 56–100		pp. 89–92		Especially pp. 68–95	Passim		
Raising the finality question								pp. 141, 280–281	pp. 27–52		
Relational connecting and fine tuning	pp. 405–504				pp. 207–223				pp. 71–98		
Tolerance for the tragic	pp. 687–713	pp. 33–58							pp. 115–130		
Compliance to emergence						pp. 42–43, pp. 80–81		p. 82–83, 99			
PERCEPTION					pp. 207–223		pp. 36–38		pp. 57–69		
Attentiveness	pp. 737–739				pp. 155–168	pp. 47–49, pp. 92–95					
Moral sensitivity	pp. 214vv pp. 692vv					p. 56 pp. 82–83					
Phenomenological sensibility	pp. 649–655					pp. 40–43 pp. 47–49					
KNOWLEDGE								pp. 53–59	Passim		
Acknowledging different sources and modes					pp. 269–289	pp. 102–106		pp. 263–264	pp. 62–63		
Critique of (discourses) of power	pp. 735–736							p. 166			
INTERPRETATION											
Experience as moral source	pp. 305–323			pp. 96–98		pp. 15–16, 56, 64–65					
Hermeneutical benevolence	pp. 80, 214, 234, 463, 627, 662, 754–757, 840–841			p. 46							
Moral deliberation	pp. 831–834			pp. 87–96					pp. 168–169		
PRACTICAL WISDOM	(pp. 819–844, 862)a					pp. 95–106		pp. 118–148	pp. 143–158	++	
Learning from the practically wise	pp. 471; 837vv								pp. 171–172	+	
Joint reflection on everyday practice	pp. 137–139					pp. 100–101				+++	
Practising of virtues	pp. 840–844								pp. 152–156		
FORMATIONAL RESOURCES			pp. 155–194						pp. 163–173		
Exposure	pp. 209–282	pp. 59–66	p. 162				pp. 77–78		pp. 163–165		pp. 20–39
Inquiry					pp. 59–89		pp. 73–76		pp. 165–166		
Deep learning									pp. 166–168		
Learning communities			pp. 167–168, 198–199		pp. 123–138, pp. 139–154		pp. 72–73		pp. 169–171		
Case-based learning							pp. 111–113		pp. 166–168		
Quality deliberation, cultivating quality awareness								Especially pp. 96–118, 237–294	pp. 177–204		
Art									pp. 172–173		

^a^Speaking about ‘normative-reflective professionality’ and ‘discretio’ as a virtue

### Exposure

Exposure is a supervised mode of exposing oneself to life in a particular situation, context, or practice, through a thorough, methodical, and intentional immersion in the situation, context or practice in question and the lifeworld of the people involved in it. The aim is to promote and facilitate a change of perspective by confronting oneself with the perspective of the people one encounters, rather than regarding everything exclusively from the perspective of one’s own frames of reference. By catching oneself in one’s responses to what is strange to oneself, and reflecting on it, one gains insight into the situation, context or practice involved, and into oneself. In the practice of relational caring as we understand it, engaging in exposure is not only a precursor to or a way of entering this practice, but also the permanent, professional, and basic attitude of the relational caring professional (Baart 2001, pp. 209–282; Baart et al. 2011, pp. 138–146; du Plessis and Beurskens 2021; Vanlaere et al. 2012, 2016).

### Inquiry

Inquiry is a continuous learning process in practice, driven by an attentive, wondering, critical and self-reflexive attitude. It is embedded in what John Dewey has called a ‘community of inquiry’ (Lipman 2003). Inquiry as we endorse combines elements of inquiry-based learning, fostering inquisitiveness (Pedaste et al. 2015), heuristic inquiry, acquiring understanding of the lived life of others (Kenny 2012; Moustakas 1990), appreciative inquiry, looking for what went well and can be helpful for the future (Cooperrider 2013), therapeutic inquiry, finding one’s freedom and open-mindedness (van der Hart et al. 2006), and moral inquiry, fostering compassion by cultivating moral imagination, especially by using art and literature (Chavel 2011; Johnson 1993; Kekes 2006). It is focused on practical wisdom. Recurrent exposures fuel this inquiry.

### Deep learning

With ‘deep learning’, we mean in-depth and long-lasting learning that affects the person themselves and their motives and intentions, their understandings and feelings. It is a kind of learning that uses real-life problem-solving in designed and supervised experiences that foster critical thinking, collaboration, character building, creativity, and citizenship. Teachers and learners engage in learning partnerships (Fullan et al. 2017). Especially working with and reflecting on one’s own experiences in life, for example by writing a diary, can be fruitful here. Training to become an ‘experience expert’ is a very advanced variant of this (Weerman and Abma 2019).

### Learning communities

Learning communities are communities of practitioners (CoPs) that aim at learning. Wenger et al. define CoPs as ‘groups of people who share a concern, a set of problems, or a passion about a topic, and who deepen their knowledge and expertise in this area by interacting on an ongoing basis’ (2002). Crucial to a CoP is a shared domain of interest, joint activities, close to the work, that make it a community, and a shared practice. Learning can be the reason the CoP comes together, or an incidental outcome of members’ interactions. Crucial to a CoP as a learning community is that learning is its aim, a learning that itself is aimed at good work. Central to a learning community in a sense that we endorse, is the free and open inquiry into people’s own everyday practising of relational caring (van Elst and Baart 2012; van Elst 2015).

### Case-based learning

A powerful way of learning is case-based learning (CBL): learning, together with colleagues and supervised by a trained tutor, about your profession by carefully scrutinizing specific cases from your own work. One of the professionals contributes a case and by interrogating the contributor, the group creates a joint image of what has actually happened. To this image belong also the logic and motives of the contributor and (the reconstruction of) the client’s own reasonableness and perspective. That ultimately leads to the question: what, after we have investigated all this, might be good to do? Essential to CBL as we advocate it, is that sufficient time is taken to jointly find out what actually happened, including the interpretations that played a role in the case, and compile a precise description before the ethical question is addressed. A session can easily take two to three hours, and sometimes turns into moral deliberation (Baart 2010; Srinivasan et al. 2007; Thistlethwaite et al. 2012).

### Cultivating quality awareness

Assessing relational caring needs to be done on the spot, in the relationships between caregivers and care receivers and in the moment. One does not need only external tools; one also must become a tool oneself, continuously and carefully inquiring in and into the concrete situation. This applies not only to the individual professional but also to teams of professionals and the organization as a whole. The cultivation of critical, vigilant quality awareness is the core of a quality policy that is able to do justice to relational caring. Cultivating quality awareness both contributes to the formation process and quality awareness itself is an outcome of such a process. Permanent quality awareness maintains the connection between learning to perceive, to understand, to appreciate and to act. It looks at the entire process, including the person counting and accounting, and thus keeps together the different ways of looking at quality and the different ways of learning. A powerful way to cultivate quality awareness is to engage in a sort of ‘quality deliberation’, a joint inquiry into care from four perspectives: of the care receiver(s), including their relatives, of the caregiver(s), of the organization, and of society (Baart 2018b; Timmerman et al. 2021).

### Art

Art, as one of the classical sources of *Bildung* (in the classical idea of it; von Bonsdorff 2012), can help cultivate free, open, and broad perception and moral imagination. And acquire a basic understanding of what it means, for example, to be lonely, excluded, vengeful, or humiliated. One can distinguish between perceiving art, perceiving *with* art, perceiving *by* art, and perceiving *as* art. Perceiving art means participating in the experience of perceiving works of art, opening oneself up to what the artist has perceived and experienced, being enriched by the artist’s knowledge and ideas. Perceiving *with* art means being criticized and corrected in the way one perceives and handles the world, by perceiving art. Perceiving *by* art means studying works of art, looking for new, alternative, disturbing perceptions, meanings, and ideas, really being changed in one’s understanding, evaluation and morals. Perceiving *as* art means making one’s own works of art as a way of doing research, letting new perceptions, understandings and meanings arise (Baart 2018a).

## Discussion

We have presented a map of core formational goals, not an educational curriculum. The map and the formational resources are based on our experience of educating, training and supervising professionals and teams of professionals in healthcare and social work (Table 1). It remains to be seen how this map can be helpful in designing a curriculum for educating students.

Can anybody learn to practise presence and relational caring? Some will say: ‘Everybody can do that and most of us do it already’, others will say: ‘It is only for the highly gifted and they do it naturally’. Based on our experience, we think, first, that there are indeed ‘virtuosos’, who practise presence and relational caring naturally. Secondly, practising presence and relational caring, even in the radical way we present here, can be learned. However, as something that must be ‘practised’, it does not always succeed. Sometimes it happens but sometimes the professional works hard and it does not happen. It depends also on the situation, the time of day, the people involved, the circumstances, et cetera. Often, learning to practise presence involves a lot of un-learning and re-learning. However, thirdly, some will never learn it, because of the kind of person they have become and especially how they have learned to deal with experiences in their own lives. Engaging in exposure is very insightful here.

Another discussion regards how practising presence can be incorporated into, or ‘mixed’ with one’s own discipline—such as nursing, medical care, social work, youth work, et cetera—and its discipline-specific theories, methods, vocabularies, and skills. We think that the practising of presence can only be (theoretically and empirically) ‘mixed’ with one’s own discipline if and to the extent that practitioners realise and acknowledge that their profession is principally a conjectural approach, not an effective procedure. Also if it has many methods at its disposal, a profession working with and for people is never simply a matter of effective procedures in which a goal can be achieved with a high degree of certainty by undertaking certain steps; rather it is a conjectural approach, in which the practitioner must do everything possible to achieve the goal but can never be sure in advance that he or she will succeed. To the extent that this is realised and acknowledged, the ‘mixing’ of practising presence with the profession involved is possible, useful, and worthwhile—even if it will not always be easy. Raising the finality question and, again, engaging in exposure are very helpful here.

Care (in healthcare and social work) is a moral practice. Educating care professionals therefore requires a reflexive relationship to ethics and to the concrete moral issues in care practices. Our proposal is situated somewhere in the middle of a spectrum between two extremes. The one extreme is a highly abstract, theoretical view of education, informed by a philosophical take on care ethics, and the other a very concrete, practical idea of training, centred around a concept of ‘applied’ care ethics. The first requires professionals to be educated in the philosophical background and principles of care ethics, the second to be trained in dealing with so-called moral dilemmas. We think both extremes fail to do justice to both actual care practices and care ethics. Neither philosophical ethics nor applied ethics can do justice to the specificity, particularity, complexity, and contingency of actual moral issues. They cannot ‘accommodate what emerges as morally relevant and even decisive in the trials and tribulations of *this* particular patient and *these* particular care professionals, within () complex () care practices’ (Vosman 2018, p. 70). The formation of relational caring professionals aims at a kind of knowing that is neither purely theoretical knowledge nor solely practical know-how, but an embodied, practical, tacit knowing ‘linked to ethically relating with particular individuals’ (formulation by one of the reviewers). This third kind of knowledge distinguishes itself from the other two by coming without claims of certainty or guarantees of success but with a commitment to persevere, acknowledging the possibility of failure. Before reducing the predicament of someone in need and the professionals who care for them to a dilemma, it is necessary to inquire into the lifeworld, the life course, the concerns, the longing, and the wishes of the participants in the situation. This requires being aware of what it is like to be poor, ill, lonely, made redundant, et cetera, as a care receiver, and to be poorly paid, placed in a straitjacket of rules and protocols, overburdened, confronted with conflicting duties, et cetera, as a care professional. Deep learning, watching movies and documentaries, reading novels and diaries, and, once again, engaging in exposures are helpful here. The question, however, is how such a formation process can be made feasible for organisations in education, and in healthcare and social work.

Lastly, there are reasons to rethink the concepts of (critical-reflexive) *Bildung* and subjectification in view of (a) the interplay between formation in the educational system, at the work place and in the student and professional’s own life, (b) the conceptualisation of care (and education) as a complex practice or form of life, i.e. an inert bundle of practices that form instances of problem-solving (Jaeggi 2019), decentring the individual professional, and (c) the inclusion of the non-human and more-than-human agencies of things, objects, materialities and spaces in our thinking about care (and education), which may demand a post-humanist concept of *Bildung* (cf. Taylor 2017).

## Conclusion

Becoming and developing oneself further as a relational caring professional requires above all a formation process as a detour of an ongoing practice. This process aims at five core domains of formational goals. Healthcare and social work should be conceptualized as a mixed practice of relational caring, with inherent moral aspects, in complex organizations in Late-Modern society. The care receiver is acknowledged as someone in a predicament, to whom caregivers should attune and from whom caregivers should not turn away. In an interplay between free, open, and careful phenomenological perception of the institutional-societal situatedness of the care receiver and knowledge from different sources, and being aware of epistemic paternalism, caregivers need to interpret the situation of the care receiver and their relatives with all its morally relevant aspects and decide what they as (political) professionals can and cannot do. Practical wisdom, learned from practically wise colleagues and from what is done well, is essential to acting in situations of uncertain outcomes, conflicting guidelines, and contradictory goals, especially in situations of complexity, ambiguity, and systemic pressure. In all these five domains, (student) caregivers need to learn and un-learn, practise and stumble, interact with care receivers and be supervised by advanced, experienced, and practically wise colleagues. The care organization’s facilitating this, especially by offering skilled facilitators and a safe environment, is essential.

## Footnotes

1 Our conceptualisation of relational caring is informed by the care-ethical theory of presence of Andries Baart (2001). In our publications in Dutch, we often use ‘presentie’ (or sometimes ‘menslievende zorg’) when talking about relational caring as we understand it.

## References

[CR1] Adams Gabrielle S, Converse Benjamin A, Hales Andrew H, Klotz Leidy E (2021). People systematically overlook subtractive changes. Nature.

[CR2] Argyris Chris, Schön Donals A (1974). Theory in practice: Increasing professional effectiveness.

[CR3] Aristotle (2013). Aristotle on practical wisdom: Nicomachean ethics VI, transl. with an introduction, analysis, and commentary by C.D.C. Reeve.

[CR4] Arvidson P. Sven (2006). The sphere of attention: Context and margin.

[CR5] Baart A (2001). Een theorie van de presentie [A theory of presence].

[CR97] Baart, A. 2004. Gebroken goed in ongebroken relaties: Theoretische notities voor de beroepspraktijk van maatschappelijke opvang [The broken good in unbroken relationships: Theoretical notes in the professional practice of social care]. In *Wanorde in een mensenleven [Disorder in a lifetime]*, ed. Marius Nuy and Frans Brinkman, 137–174. Amsterdam: SWP.

[CR98] Baart, A. 2008. Over de verdringing van praktijkkennis [About the supplanting of practical wisdom]. In *Aannemelijke zorg [Plausible care]*, ed. Frans Vosman and Andries Baart, 49–134. Utrecht: Lemma.

[CR99] Baart, A. 2010. Case-based learning in zeven stappen [Case-based learning in seven steps]. In *Zwerfwerken [Homeless work]*, ed. Karin Runia and Roelof Hortulanus, 151–152. Utrecht: Lesi. 10.13140/RG.2.2.15282.50887.

[CR100] Baart, A. 2011. *Van bewegen naar bewogenheid: Een fenomenologische verkenning van zorg geven in een politiek-ethisch perspectief [From moving to compassion: A phenomenological exploration of giving care in a political-ethical perspective]*. Amsterdam: SWP.

[CR6] Baart, A. 2013. De zorgval: Analyse, kritiek en uitzicht [The care trap: Analysis, critique and outlook]. In *De zorgval [The care trap]*, ed. A. Baart and C. Carbo, 5–128. Amsterdam: Thoeris.

[CR101] Baart, A. 2014. *The formation of the care ethical practitioner*. 10.13140/RG.2.2.16069.96487.

[CR102] Baart, A. 2018a. *De kunst van het waarnemen en het waarnemen van de kunst [The art of perceiving and perceiving art]*, lecture at Sympósion 17 April 2018. https://vimeo.com/273004704.

[CR7] Baart, A. 2018b. *De ontdekking van kwaliteit: Theorie en praktijk van relationeel zorg geven [The discovery of quality: Theory and practice of relational care]*. Amsterdam: SWP.

[CR103] Baart, A. 2020. Empirically grounded ethics of care: An argument. In *The ethics of care: The state of the art*, ed. Frans Vosman, Andries Baart and Jaco Hoffman, 261–298. Leuven: Peeters.

[CR104] Baart, A. 2021. Precariousness, precarity, precariat, precarization and social redundancy: A substantiated map for the ethics of care. In *Care ethics in the age of precarity*, ed. Maurice Hamington and Michael Flower, 91–119. Minneapolis MN: University of Minnesota Press.

[CR8] Baart, A., and M. Grypdonck. 2008. *Verpleegkunde en presentie: Een zoektocht in dialoog naar de betekenis van presentie voor verpleegkundige zorg [Nursing and presence: A search in dialogue for the meaning of presence for nursing]*. Den Haag: LEMMA.

[CR105] Baart, A., and F. Vosman. 2011. Relationship based care and recognition: Part one: Sketching good care from the theory of presence and five entries. In *Care, compassion and recognition*, ed. Carlo Leget, Chris Gastmans and Marian Verkerk, 183–200. Leuven: Peeters.

[CR9] Baart, A., J. van Dijke, M. Ouwerkerk, and E. Beurskens. 2011. *Buigzame zorg in een onbuigzame wereld: Presentie als transitiekracht [Flexible care in an inflexible world: Presence as a strength for transition]*. Den Haag: Lemma.

[CR10] Baart, A., F. Vosman, et al. 2015. *De patiënt terug van weggeweest: Werken aan menslievende zorg in het ziekenhuis [The comeback of the patient: Working on professional loving care in hospitals]*. Amsterdam: SWP.

[CR11] Barnes Marian (2012). Care in everyday life: An ethic of care in practice.

[CR12] Benard Bonnie (2004). Resiliency: What we have learned.

[CR13] Benner Patricia (1994). Interpretive phenomenology: Embodiment, caring, and ethics in health and illness.

[CR14] Beurskens, E., M. van der Linde, and A. Baart. 2019. *Praktijkboek presentie [Practising presence]*. Bussum: Coutinho.

[CR15] Biesta Gert (2020). Risking ourselves in education: Qualification, socialization, and subjectification revisited. Educational Theory.

[CR16] Bontemps-Hommen, Marij. 2020. *Practical wisdom: The vital core of professionalism in medical practices*. Doctoral dissertation University of Humanistic Studies, Afferden. https://research.uvh.nl/en/publications/practical-wisdom-the-vital-core-of-professionalism-in-medical-pra.

[CR17] Bontemps-Hommen MCMML, Baart AJ, Vosman FJH (2020). Professional workplace-learning. Can practical wisdom be learned?. Vocations and Learning.

[CR18] Brister Evelyn (2012). Distributing epistemic authority: Refining Norton’s pragmatist approach to environmental decision-making. Contemporary Pragmatism.

[CR19] Chavel Solange (2011). Se mettre à la place d’autrui: L’imagination morale [Put yourself in the shoes of others: Moral imagination].

[CR20] Cooperrider David L, Cooperrider David L, Zandee Danielle P, Godwin Lindsey N, Avital Michel, Boland Brodie (2013). A contemporary commentary on appreciative inquiry in organizational life. Organizational generativity.

[CR21] Daaleman Timothy P, Kinghorn Warren A, Newton Warren P, Meador Keith G (2011). Rethinking professionalism in medical education through formation. Family Medicine.

[CR22] Dancy Jonathan, Zalta Edward N (2017). Moral particularism. The Stanford encyclopedia of philosophy.

[CR23] de Sousa Santos Boaventura (2018). The end of the cognitive empire: The coming of age of epistemologies of the South.

[CR24] du Plessis, E., and E. Beurskens. 2021. A deeper understanding of presence through an exposure. In *Reflecting on presence in nursing: A guide for practice and research*, ed. E. du Plessis, 20–39. Newcastle upon Tyne: Cambridge Scholars.

[CR26] Engster Daniel (2007). The heart of justice: Care ethics and political theory.

[CR27] Foucault, Michel. 2011. *The courage of the truth (The government of self and others II): Lectures at the Collège de France 1983–1984]*, ed. Frédéric Gros, trans. Graham Burchell. New York: Palgrave Macmillan.

[CR28] Foucault Michel (2001). Fearless speech.

[CR29] Fricker Miranda (2007). Epistemic injustice: Power and the ethics of knowing.

[CR30] Fullan Michael, Quinn Joanne, McEachen Joanne (2017). Deep learning: Engage the world change the world.

[CR31] Gadamer Hans-Georg (1994). Truth and method, trans. rev. Joel Weinsheimer and Donald G. Marshall.

[CR32] Greve Jens, Schnabel Annette (2011). Emergenz: Zur Analyse und Erklärung Komplexer Strukturen.

[CR33] Grøthe Å, Biong Stian, Grov EK (2015). Acting with dedication and expertise: Relatives’ experience of nurses’ provision of care in a palliative unit. Palliative and Supportive Care.

[CR35] Housset Emmanuel (2003). L’intelligence de la pitié: Phénoménology de la communauté [The intelligence of mercy: Phenomenology of community].

[CR36] Jacobs Gabu, Meij Ruud, Tenwolde Hans, Zomer Yanaika (2008). Goed werk: Verkenningen van normatieve professionalisering [Good work: Explorations of normative professionalization].

[CR37] Jaeggi Rahel (2019). Critique of forms of life, trans. Ciaran Cronin.

[CR38] Johnson Mark (1993). Moral imagination: Implications of cognitive science to ethics.

[CR39] Kekes John (2006). The enlargement of life: Moral imagination at work.

[CR40] Kenny Gerard (2012). An introduction to Moustakas’s heuristic method. Nurse Researcher.

[CR41] Kinsella Elizabeth Anne, Pitman Allen (2012). Phronesis as professional knowledge: Practical wisdom in the professions.

[CR42] Klafki Wolfgang (2007). Neue Studien zur Bildungstheorie und Didaktik: Zeitgemäße Allgemeinbildung und kritisch-konstruktive Didaktik [New studies on educational theory and didactics: Contemporary general education and critical-constructive didactics].

[CR43] Klaver, Klaartje. 2016. *Dynamics of attentiveness: In care practices at a Dutch oncology ward*. Doctoral dissertation Tilburg University. https://pure.uvt.nl/portal/files/10987382/Klaver_Dynamics_01_04_2016.pdf.

[CR106] Klaver, Klaartje, and Andries Baart. 2011. Attentiveness in care: Towards a theoretical framework. *Nursing Ethics* 18 (5): 686–693. 10.1177/0969733011408052 10.1177/0969733011408052 21788284

[CR107] Klaver, Klaartje, Eric van Elst, and Andries J. Baart. 2014. Demarcation of the ethics of care as a discipline. *Nursing Ethics* 21(7): 755–765. 10.1177/0969733013500162 10.1177/0969733013500162 24154572

[CR44] Kuokkanen Liisa, Leino-Kilpi Helena (2001). The qualities of an empowered nurse and the factors involved. Journal of Nursing Management.

[CR45] Laros Anna, Taylor Thomas Fuhr an Edward W. (2017). Transformative learning meets Bildung: An international exchange.

[CR46] Leest Judith, Bolt Eveline, van der Linde Marije (2018). Verstandig duikelen: De complexe praktijk van sociale professionals [Tumbling sensibly: The complex practice of social workers].

[CR48] Leget Carlo, van Nistelrooij Inge, Visse Merel (2017). Beyond demarcation: Care ethics as an interdisciplinary field of inquiry. Nursing Ethics.

[CR49] LeVasseur Jeanne J (2003). The problem of bracketing in phenomenology. Qualitative Health Research.

[CR50] Lindblom Charles E (1959). The science of muddling through. Public Administration Review.

[CR51] Lindblom Charles E (1979). Still muddling, not yet through. Public Administration Review.

[CR52] Lipman Matthew (2003). Thinking in education.

[CR53] Loorbach Derk, Rotmans Jan (2010). The practice of transition management: Examples and lessons from four distinct cases. Futures.

[CR54] Morin, Edgar. 2008. *On complexity*, trans. Robin Postel and Sean M. Kelly. Chesskill NJ: Hampton.

[CR55] Moustakas Clark (1990). Heuristic research: Design, methodology, and applications.

[CR56] Nicolini Davide (2012). Practice theory, work, and organization: An introduction.

[CR57] Nicolini Davide, Monteiro Pedro, Langley Ann, Tsoukas Haridimos (2017). The practice approach: For a praxeology of organisational and management studies. Handbook of process organization studies.

[CR58] Nussbaum Martha C (2001). Upheavals of thought: The intelligence of emotions.

[CR59] Nussbaum Martha (2004). Emotions as judgments of value and importance. Thinking about feeling: Contemporary philosophers on emotions.

[CR60] Nussbaum Martha C (2013). Political emotions: Why love matters for justice.

[CR108] Olthuis, Gert, Carolien Prins, Marie-Josée Smits, Harm van de Pas, Joost Bierens, and Andries Baart. 2014. Matters of concern: A qualitative study of emergency care from the perspective of patients. *Annals of Emergency Medicine* 63(3): 311–319. 10.1016/j.annemergmed.2013.08.018 10.1016/j.annemergmed.2013.08.018 24054787

[CR61] Pedaste Margus, Mäeots Mario, Siiman Leo A, de Jong Ton, van Riesen Siswa A.N., Kamp Ellen T, Manoli Constantinos C, Zacharia Zacharias C, Tsourlidaki Eleftheria (2015). Phases of inquiry-based learning: Definitions and the inquiry cycle. Educational Research Review.

[CR62] Polanyi Michael (1958). Personal knowledge: Towards a post-critical philosophy.

[CR63] Polanyi Michael (1966). The tacit dimension.

[CR64] Reichenbach Roland (2014). Humanistic Bildung: Regulative idea or empty concept?. Asia Pacific Education Review.

[CR65] Roeser Sabine (2011). Moral emotions and intuitions.

[CR66] Sayer Andrew (2011). Why things matter to people: Social science, values and ethical life.

[CR67] Schatzki Theodore R (1996). Social practices: A Wittgensteinian approach to human activity and the social.

[CR68] Schön Donald (1983). The reflective practitioner.

[CR69] Schön Donald (1987). Educating the reflective practitioner.

[CR70] Schwartz Barry, Sharpe Kenneth (2010). Practical wisdom: The right way to do the right thing.

[CR71] Senge Peter, Jaworski Joseph, Scharmer Otto, Flowers Betty Sue (2005). Presence: Exploring profound change in people, organizations and society.

[CR72] Sennett Richard (2008). The craftsman.

[CR73] Sevenhuijsen Selma, Sevenhuijsen Selma, Švab Alenka (2004). Trace: A method for normative policy analysis from the ethic of care. The heart of the matter.

[CR74] Sjöström Jesper, Frerichs Nadja, Zuin Vânia, Eilks Ingo (2017). Use of the concept of Bildung in the international science education literature, its potential, and implications for teaching and learning. Studies in Science Education.

[CR75] Srinivasan M, Wilkes M, Stevenson F, Nguyen T, Slavin S (2007). Comparing problem-based learning with case-based learning: Effects of a major curricular shift at two institutions. Academic Medicine.

[CR76] Taylor Charles (2007). Modern social imaginaries.

[CR77] Taylor Carol A (2017). Is a posthumanist *Bildung* possible? Reclaiming the promise of *Bildung* for contemporary higher education. Higher Education.

[CR78] Thistlethwaite Jill Elizabeth, Davies David, Ekeocha Samilia, Kidd Jane M, MacDougall Colin, Matthews Paul, Purkis Judith, Clay Diane (2012). The effectiveness of case-based learning in health professional education. A BEME systematic review: BEME guide no. 23. Medical Teacher.

[CR79] Timmerman, G., and A. Baart. 2016a. Ongeregeld goed: De huisarts aan het ziek- en sterfbed van de eigen patiënt [Unregulated good: The general practitioner at the bedside of their sick and dying patient]. *Stichting Presentie, Utrecht.*10.13140/RG.2.2.17048.83203.

[CR109] Timmerman, G., and A. Baart. 2016b. Präsentische praxis und die theorie der präsenz [practice and theory of presence]. In *Praxis der Achtsamkeit [Practice of care]*, ed. Elisabeth Conradi and Frans Vosman, 189–208. Frankfurt: Campus.

[CR110] Timmerman, Guus, Andries Baart, and Jan den Bakker. 2021. Cultivating quality awareness in corona times. *Medicine Health Care and Philosophy* 24(2): 189–204. 10.1007/s11019-021-10010-x. 10.1007/s11019-021-10010-x PMC8009932 33788079

[CR81] Tronto Joan C (1993). Moral boundaries: A political argument for an ethic of care.

[CR111] van Elst, Eric. 2015. ‘Poulet à la d’albufera’ of hoe bereid je een bloeiende lerende gemeenschap [‘Poulet à la d’albufera’ or how do you prepare a thriving learning community]. In *De patiënt terug van weggeweest [The comeback of the patient]*. Andries Baart, Frans Vosman et al., 123–138. Amsterdam: SWP.

[CR82] van der Hart, Onno, Ellert R.S.. Nijenhuis, and Kathy Steele. 2006. *The haunted self: Structural dissociation and the treatment of chronic traumatization*. New York: W.W. Norton & Company.

[CR112] van Elst, Eric, and Andries Baart, A. 2012. Hoezo implementeren? Anders leren! [Implementing? Learning differently!]. In *Menslievende zorg in de praktijk [Professional loving care in practice]*, ed. Carlo Leget and Gert Olthuis, 79–88. Den Haag: Lemma.

[CR83] van Ewijk Hans, Kunneman Harry (2013). Praktijken van normatieve professionalisering [Practices of normative professionalization].

[CR34] van Heijst, Annelies. 2011. *Professional loving care: An ethical view of the health care sector*, trans. Kay Caldwell. Leuven: Peeters.

[CR84] van Tongeren Paul (1994). Narrativiteit en hermeneutiek in verband met een adequate praktische ethiek [Narrativity and hermeneutics in connection with an adequate practical ethics]. Ethische Perspectieven.

[CR80] van Tongeren, Paul. 1999. Ethiek als hermeneutiek van de morele ervaring [Ethics as hermeneutics of moral experience]. In *Ethiek en hermeneutiek*, ed. Jean-Pierre Wils, 217–225. Leende: Damon.

[CR85] Vanlaere, Linus, Madeleine Timmermann and Mieke Grypdonck. 2016. Pflegehandeln am eigenen Körper erfahren: ‘Ausgesetzsein’ in simulierten Situationen [Experiencing nursing on one’s own body: ‘Being exposed’ in simulated situations]. In *Praxis der Achtsamkeit*, ed. Elisabeth Conradi and Frans Vosman, 231–247. Frankfurt: Campus.

[CR86] Vanlaere Linus, Timmermann Madeleine, Stevens Leen, Gastmans Chris (2012). An explorative study of experiences of healthcare providers posing as simulated care receivers in a ‘care-ethical’ lab. Nursing Ethics.

[CR87] Verhoeven, Cornelis. 1972. *The philosophy of wonder: An introduction and incitement to philosophy*, trans. Mary Foran. New York: MacMillan.

[CR88] von Bonsdorff Pauline (2012). Aesthetics and bildung. Diogenes.

[CR89] Vosman, Frans. 2008. Over het uitzieden van praktische wijsheid [About distilling practical wisdom]. In *Aannemelijke zorg [Plausible care]*. Dual inaugural lecture Tilburg University, ed. Frans Vosman and Andries Baart (pp. 11–47). Den Haag: LEMMA.

[CR90] Vosman, Frans. 2018. The moral relevance of lived experience in complex hospital practices: A phenomenological approach. In *Theological ethics and moral value phenomena*, ed. Steven C. van den Heuvel, Paul Nullens, and Anegla Roothaan, 65–92. New York: Routledge.

[CR91] Vosman Frans, Vosman Frans, Baart Andries, Hoffman Jaco (2020). The disenchantment of care ethics: A critical cartography. The ethics of care: The state of the art.

[CR113] Vosman, Frans, and Andries Baart. 2008. *Aannemelijke zorg: Over het uitzieden en verdringen van praktische wijsheid in de gezondheidszorg [Plausible care: About distilling and supplanting practical wisdom in healthcare]*. Den Haag: Lemma.

[CR92] Vosman, Frans, Jan den Bakker, and Don Weenink. 2016. How to make sense of suffering in complex care practices? In *Practice theory and research*, ed. Gert Spaargaren, Don Weenink, and Machiel Lamers, 117–130. New York: Routledge.

[CR93] Vosman Frans, Niemeijer Alistair (2017). Rethinking critical reflection on care: Late modern uncertainty and the implications for care ethics. Medicine, Health Care and Philosophy.

[CR114] Vosman, Frans, Guus Timmerman, and Andries Baart. 2018. Digging into care practices: the confrontation of care ethics with qualitative empirical and theoretical developments in the Low Countries 2007–17. *International Journal of Care and Caring* 2(3): 405–423. 10.1332/239788218X15321005652967.

[CR94] Waldenfels, Bernhard. 2004. *Phänomenologie der Aufmerksamkeit* [Phenomenology of attentiveness]. Frankfurt: Suhrkamp.

[CR95] Weerman Alie, Abma Tineke (2019). Social work students learning to use their experiential knowledge of recovery: An existential and emancipatory perspective. Social Work Education.

[CR96] Wenger Etienne, McDermott Richard, Snyder William M (2002). Cultivating communities of practice: A guide to managing knowledge.

